# Improved Production of Active *Streptomyces griseus* Trypsin with a Novel Auto-Catalyzed Strategy

**DOI:** 10.1038/srep23158

**Published:** 2016-03-17

**Authors:** Yunfeng Zhang, Zhenmin Ling, Guocheng Du, Jian Chen, Zhen Kang

**Affiliations:** 1 Key Laboratory of Industrial Biotechnology, Ministry of Education, Jiangnan University, 1800 Lihu Road, Wuxi, Jiangsu 214122, China; 2 Synergetic Innovation Center of Food Safety and Nutrition, 1800 Lihu Road, Wuxi, Jiangsu 214122, China; 3 The Key Laboratory of Carbohydrate Chemistry and Biotechnology, Ministry of Education, School of Biotechnology, Jiangnan University, Wuxi 214122, China

## Abstract

N-terminal sequences play crucial roles in regulating expression, translation, activation and enzymatic properties of proteins. To reduce cell toxicity of intracellular trypsin and increase secretory expression, we developed a novel auto-catalyzed strategy to produce recombinant trypsin by engineering the N-terminus of mature *Streptomyces griseus* trypsin (SGT). The engineered N-terminal peptide of SGT was composed of the thioredoxin, glycine-serine linker, His_6_-tag and the partial bovine trypsinogen pro-peptide (DDDDK). Furthermore, we constructed a variant TLEI with insertion of the artificial peptide at N-terminus and site-directed mutagenesis of the autolysis residue R145. In fed-batch fermentation, the production of extracellular trypsin activity was significantly improved to 47.4 ± 1.2 U·ml^−1^ (amidase activity, 8532 ± 142.2 U·ml^−1^ BAEE activity) with a productivity of 0.49 U·ml^−1^·h^−1^, which was 329% greater than that of parent strain *Pichia pastoris* GS115-SGT. This work has significant potential to be scaled-up for microbial production of SGT. In addition, the N-terminal peptide engineering strategy can be extended to improve heterologous expression of other toxic enzymes.

Trypsin (EC 3.4.21.4), a serine protease, is used in leather bating, food processing, pharmaceuticals, clinical diagnoses and biochemical testing applications^1^,^2^. Currently, trypsin has been discovered in many mammalian and bacterial sources, and the trypsin extracted from bovine pancreas is a well-known commercial product. However, this conventional approach of producing trypsin from bovine pancreas cannot meet the high demand, and may be contaminated with pathogens or viruses^3^,^4^, which poses as a potential hazard to mammal immunogenicity when used in the pharmaceutical industry. Therefore, to circumvent these problems, trypsinogens originating from various organisms are heterologously expressed in different hosts. When trypsinogen is expressed in an *Escherichia coli* system, the majority of the over-produced protein is found in inclusion bodies^5^. Although bovine and shrimp trypsinogen are expressed in soluble form by *Pichia pastoris*, its activation is dependent on digestion with enterokinase^6^,^7^. As alternatives, the bovine trypsin gene is also studied in transgenic rice cells^8^ and *Lactococcus lactis*^9^; however, the production of active recombinant trypsin is low even after codon optimization. As a result, it is always a big challenge to efficiently produce active trypsin with microbial cell factories.

As a potential alternative candidate, *Streptomyces griseus* trypsin (SGT), which is composed of 223 residues with a molecular weight of 23.1 kDa, has 34.2% homology with bovine trypsin in amino acid sequence, including the active site residues (H57, D102 and S195), substrate binding region and substrate specificity^10^,^11^,^12^. Moreover, compared with bovine trypsin (BT), SGT possesses only three pair of disulfide bonds and higher catalytic efficiency (i.e., *k*_cat_/*K*_m_ Table 1). As a result, the SGT encoding gene has been cloned and expressed in *S. lividans*^13^,^14^, *S. griseus*^15^ and *Bacillus subtilis*^16^. Recently, we realized its secretory and active expression in *P. pastoris*^17^. Furthermore, the results also confirmed previous hypothesis that activation of the SGT in *S. griseus* is not self-activated^1^. However, the detailed activation mechanism remains unresolved^18^. In comparison, bovine trypsinogen is activated by enterokinase cleavage at the P_1_ site (Lys) of the recognized pro-peptide VDDDDK (VD4K)^19^ or itself^20^,^21^,^22^,^23^,^24^. Although substitution of the native pro-peptide APNP with VD4K resulted in expression of SGT in inactive form, we found that the soluble hybrid trypsinogen could be activated following removal of VD4K by enterokinase *in vitro*, suggesting that the partial bovine trypsinogen pro-peptide VD4K could assist in expressing the SGT in trypsinogen form for avoiding intracellular toxicity to host^17^. By combining *in silico* simulations and the results *in vitro* experiments, several variants with different short pro-peptides have been recently constructed and used for improving SGT production in *P. pastoris*^25^. Similarly, several studies aimed at improving the production of target enzymes with engineered N-terminal regions have been reported in *E. coli* and *P. pastoris*^26^,^27^,^28^,^29^. Therefore, the combination of N-terminal engineering and other internal modification methods such as site-directed mutagenesis may represent an efficient and practical approach for improving the production and enzymatic properties of target enzymes.

In the present study, we have developed a novel self-activation strategy to decrease the protease toxicity to cells and improve the titer and enzymatic properties of SGT. Applying *in silico* simulations and *in vitro* experiments we rationally engineered the N-terminus of SGT with differently artificial peptides (Fig. 1). Enzyme activity and N-terminal sequencing results demonstrate that the rationally designed SGT variant achieved secretory expression with a self-activation pattern. In fed-batch fermentation (3 l fermenter), the titer of recombinant SGT was significantly enhanced from 14.4 U·ml^−1^ 17 to 47.4 ± 1.2 U·ml^−1^ with 329% increase.

## Results and discussion

### Effect of the pro-peptide D4K on SGT active expression

*Streptomyces griseus* trypsinogen consists of two segments: a pro-peptide and SGT. This structural architecture is similar to the domain arrangement of bovine trypsinogen (Supplementary Fig. S1). Nonetheless, the activation mechanism of SGT remains unresolved and is likely distinct from that of bovine trypsin, because the SGT was extracellularly expressed in inactive form after replacement of the SGT pro-peptide (APNP) with the partial bovine trypsinogen pro-peptide^17^. Currently, *in vitro* investigations combining *in silico* simulations with experiments are common approaches for studying the relationship between enzyme properties and structural changes^30^. Accordingly, the structure of the variant D4K-SGT with pro-peptide D_4_K at N-terminal was simulated to reveal the influence of the pro-peptide D_4_K on the conformation of SGT (PDB ID: 1SGT). The hybridized partial bovine trypsinogen pro-peptide D_4_K was found to be buried in D4K-SGT, which caused a loosened tertiary structure comparing with SGT (Fig. 2A). Moreover, the disulfide bonds (C42-C58, C168-C182, C191-C220) were mismatched in D_4_K-SGT (α. 2B) and an inaccurate catalytic triad conformation formed because of the changed hydrogen bonds (with disruption of the hydrogen bonds between D102 and H57) and substrate binding pocket (Fig. 2C), eventually resulting in an inactive state. The results also suggest that it would be attractive to release the bound peptide to give self-activation during the process of secretion, because overexpression of the active form of SGT is toxic to the host strain^17^.

### Secretory production of active trypsin with a self-activation approach

**Figure 1:**
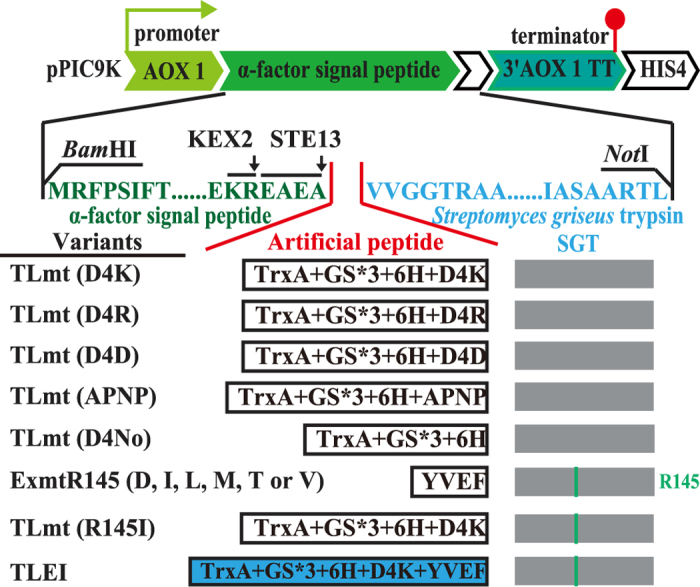
Schematic representation of the vector construction and the trypsin variants. The artificial peptide segment of TLmt (D4K) is composed of TrxA (thioredoxin), GS*3 (GSGSGS, glycine-serine linker), 6H (His6-tag) and D4K (DDDDK, the partial bovine trypsinogen pro-peptide). In variants TLmt (D4R), TLmt (D4D) and TLmt (APNP), the pro-peptide D4K were substituted with DDDDR, DDDDD, DDDDD and APNP, respectively. Exmt represent a variant with the inserted peptide YVEF at N-terminus of SGT^25^.

**Figure 2:**
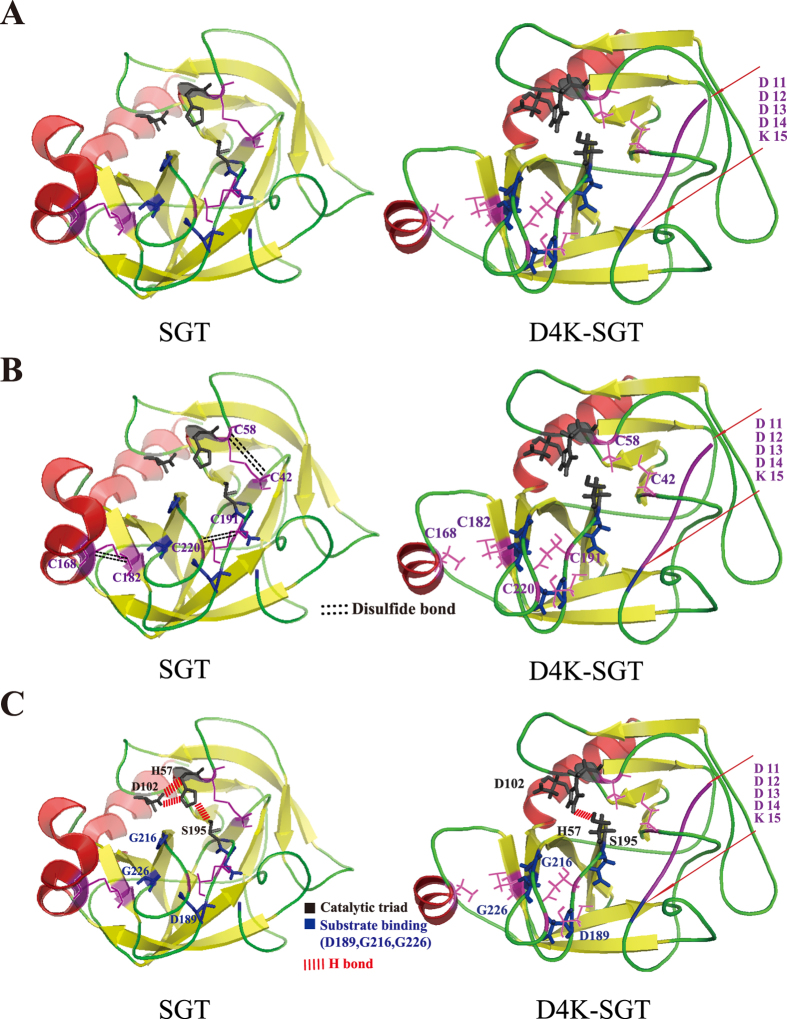
*In silico* simulation and analysis of the D4K-SGT. (**A**) Tertiary structure comparison of SGT and D4K-SGT. (**B**) The disulfide bonds comparison of SGT and D4K-SGT. (**C**) The catalytic triad and substrate banding pocket comparison of SGT and D4K-SGT.

**Figure 3:**
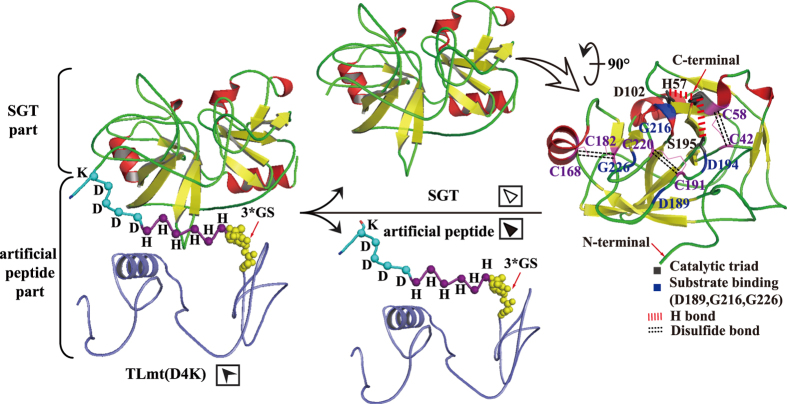
*In silico* simulation and illustration of the self-activation process of the variant TLmt (D4K). The artificial peptide of TLmt (D4K) was released from the mature SGT, with readily recognizing and cleaving at pro-peptide D4K.

To realize the auto-cleavage of the embedded artificial peptide and the activation of SGT, a number of N-terminal peptides were designed and fused to the N-terminus of SGT to generate SGT variants (Fig. 1). Specifically, TrxA (encoded by the *trxA* gene), which functions as a chaperone for assisting folding and accumulation of heterologous proteins^31^,^32^,^33^,^34^ was fused between the C-terminus of the α-factor signal peptide and the N-terminus of SGT. Moreover, a glycine-serine (GS*3) linker followed by a His_6_-tag (for facilitating purification of TrxA protein) was designed and inserted to avoid TrxA affecting the tertiary conformation of SGT. To achieve self-activation with elimination of the N-terminal peptides, the partial native bovine pro-peptide D4K (can be facilely recognized and cleaved by the recombinant SGT) and other pro-peptides were designed and comparatively investigated (Fig. 1). Accordingly, three dimensional structure of the variant TLmt (D4K) was simulated to evaluate our hypothesis. As shown in Fig. 3, the artificial peptide of TLmt (D4K) was outside of the mature SGT, which would be readily recognized and cleaved. In addition, no obvious alterations to the tertiary conformation of SGT (PDB ID: 1SGT) were observed, indicating active SGT could be released after autocatalysis.

**Figure 4:**
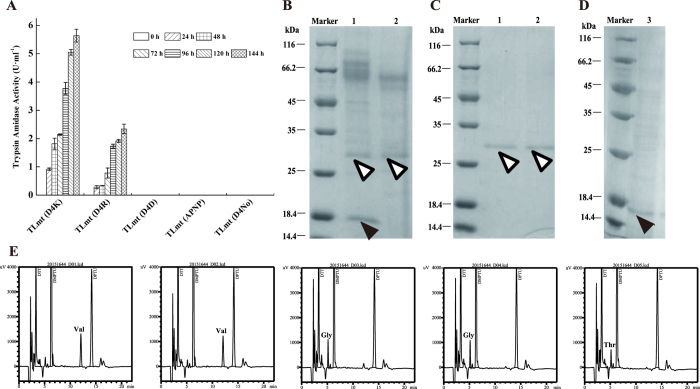
Comparison of the variants with different pro-peptides. (**A**) Trypsin amdidase activity of the variants with different pro-peptides, with 1% (v/v) methanol inducing for 144 h in flasks. (**B**) SDS-PAGE results of the culture supernatant of GS115-TLmt (D4K) variant (1) and GS115-SGT (2). (**C**) SDS-PAGE results of the purified active SGT from culture supernatant of GS115-TLmt (D4K) variant (1) and GS115-SGT (2). (**D**) SDS-PAGE result of the purified TrxA (3) from culture supernatant of GS115-TLmt (D4K) variant. Active SGT (Δ) and TrxA (▲). (**E**) The N-terminal five amino acids of the purified active SGT from TLmt (D4K).

To verify the above simulation results, all the recombinant strains containing the same copy numbers of variant *rSGT* genes were cultivated in flasks (Supplementary Fig. S2A). Notably, the variants TLmt (D4K) and TLmt (D4R) were successfully secreted in the active form. After induction for 144 h with 1% methanol (v/v), the trypsin enzyme activity (amidase) of TLmt (D4K) was 1.58 times higher than that of TLmt (D4R) (Fig. 4A), suggesting that the pro-peptide D4K was more readily recognized and catalyzed when compared with the D4R sequence. In contrast, no trypsin activity was detected in the cultures of the other variants, suggesting the catalytic specificity of SGT to amino acid residuals K or R.

To further verify the above results, the extracellular culture supernatant of the strain GS115-TLmt (D4K) was analyzed by SDS-PAGE with GS115-SGT as a control^35^. As shown in Fig. 4B–D, the bands corresponding to the active SGT (~ 28 kDa) and TrxA (~18 kDa) were clearly observed. Moreover, N-terminal sequencing results showed that the secreted active SGT shared the same initial five amino acids (VVGGT) (Fig. 4E) compared to the original sequence of 1SGT (PDB ID). Taken together, the results demonstrate that it is feasible and desirable to achieve self-activation of SGT by introducing artificial peptide.

### Modification of the autolysis sensitive site R145 to improve the stability

**Figure 5:**
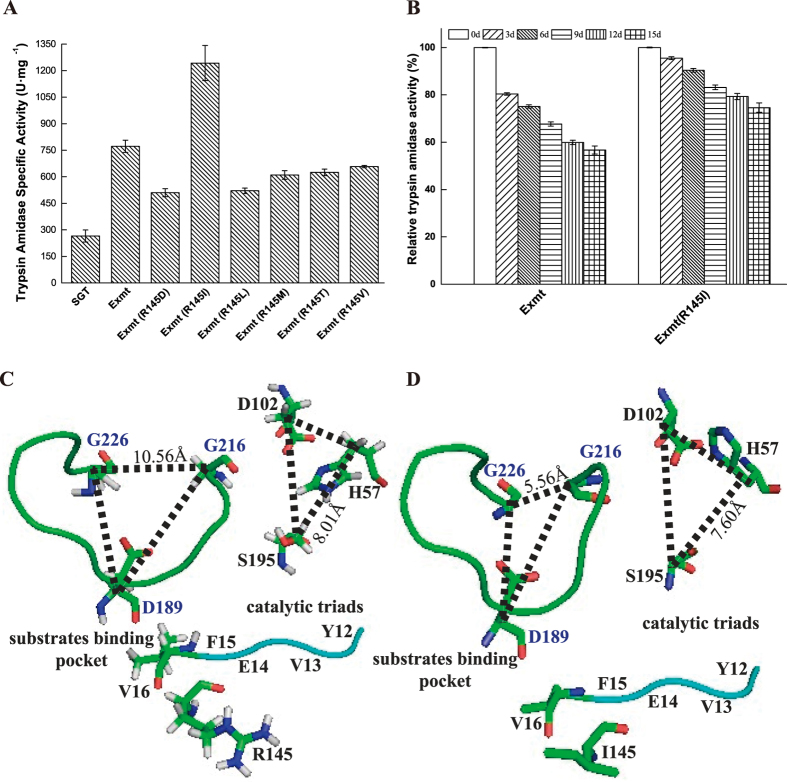
Comparison of the different variants with the engineered R145 residue. (**A**) Trypsin amidase specific activity of Exmt (R145 mutations). (**B**) Relative trypsin amidase activity of h and recombinant SGT. (**C**) and (**D**) are the simulated catalytic center structures of the variants Exmt and Exmt (R145I), respectively.

In SGT, the autolysis loop (residues 141–152) has been reported to be flexible region and the primary target for autolysis^36^. In particular, the sensitive autolysis site R145 is recognized and cleaved by active trypsin^37^. Consequently, desensitization of the autolysis loops, especially the sensitive site, provide greater stability. As shown in Fig. 1, according to the simulation results (online sever http://dezyme.com/)^38^, six forward variants of Exmt (a variant of SGT with an inserted N-terminal peptide YVEF^25^) were constructed. The strains that contained the Exmt (R145 mutations) encoding gene with 9 copies (Supplementary Fig. S2B) were cultivated in flasks by inducing with 1% (v/v) methanol for 144 h. Then, the supernatant of each culture was purified for measuring specific activity to two substrates BAPNA and BAEE (Supplementary Fig. S3). Significantly, the mutant Exmt (R145I) showed the highest amidase specific activity (1,242.85 ± 99.15 U·mg^−1^) (Fig. 5A) and the highest esterase specific activity (101,491.58 ± 1,225.56 U·mg^−1^) (Supplementary Fig. S4), indicating the higher catalytic efficiency of the mutant Exmt (R145I). Subsequently, kinetic parameters of all the variants were further determined and compared. Although a decreased substrate affinity (i.e., *K*_m_ toward BAPNA) was observed, the variant Exmt (R145I) showed the highest catalytic efficiency (i.e., *k*_cat_/*K*_m_) towards BAPNA (Table 1). The reason should be ascribed to the decreased Cα distance between S195 and H57 in catalytic triad (which benefits electron transportation)^39^,^40^ even with a narrowed distance between G216 and G226 in substrate binding pocket (Fig. 5C,D). Moreover, it could be found that Exmt (R145I) remained higher amidase activity than Exmt (Fig. 5B) after 15 days. In addition, both SDS-PAGE (Supplementary Fig. S5A,B) and molecular dynamics simulation RMSD (root mean square deviation) analysis results (Supplementary Fig. S6) also demonstrated that introduction of the mutation R145I improved the stability against self-autolysis.

### Construction and fed-batch fermentation of the self-activation variants without autolysis

**Figure 6:**
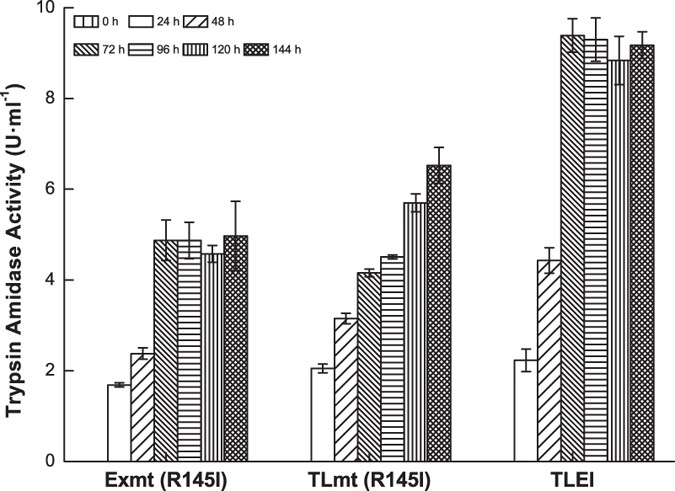
Trypsin amidase activity of the recombinants GS115-Exmt (R145I), GS115-TLmt (R145I) and GS115-TLEI.

According to above results, N-terminal engineering gave the variant TLmt (D4K) secretory expression with a self-activation strategy that could be beneficial in reducing the protease toxicity of trypsin to the host strain. Simultaneously, site-directed mutagenesis of R145 to I significantly improved the stability and the catalytic efficiency. As a result, we further constructed the variants TLmt (R145I) and TLEI (Fig. 1), and comparatively investigated the trypsin activity with Exmt (R145I). As shown in Fig. 6, after 1% (v/v) methanol inducing for 144h, the amidase activity in the extracellular culture supernatant of the strains GS115-Exmt (R145I), GS115-TLmt (R145I) and GS115-TLEI were 4.97 ± 0.76, 6.53 ± 0.39 and 9.39 ± 0.36 U·ml^−1^, respectively, whereas no intracellular activity was detected.

**Figure 7:**
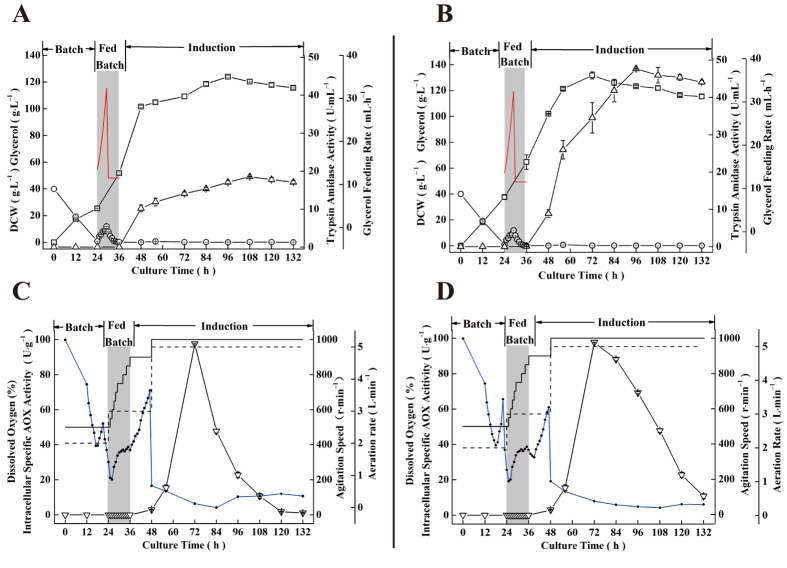
Time course profile of recombinant trypsin production in an 3 l fermenter. (**A,C**) of GS115-Exmt (R145I); (**B,D**) of GS115-TLEI. Trypsin activity (△), dry cell weight (◻), residual glycerol (○), glycerol feeding rate (^__^), dissolved oxygen (●), intracellular specific AOX activity (▽), agitation speed (^__^) and aeration rate (---).

To evaluate the trypsin production capacity of the strain GS115-TLEI, fed-batch fermentation with GS115-Exmt (R145I) as the control was further carried out. As shown in Fig. 7, the operation process was composed of three parts according to previous study^41^. Specifically, no trypsin was found to accumulate in the culture before induction. When methanol was added as the inducer, the amount of extracellular recombinant trypsin was rapidly accumulated. At 108 h, the amidase activity in supernatant of the GS115-Exmt (R145I) culture was increased to 18.4 ± 0.8 U·ml^−1^ (Fig. 7A). In comparison, the amidase activity in supernatant of the GS115-TLEI culture was accumulated to 47.4 ± 1.2 U·ml^−1^ (corresponding to 8532 ± 142.2 U·ml^−1^ esterase activity) at 96 h (Fig. 7B). Moreover, it could be found that compared with GS115-Exmt (R145I) the GS115-TLEI showed better cell growth and the biomass was accumulated to 130 g·l^−1^ (DCW) at 72 h, indicating the release of protease toxicity of the variant TLEI^42^. In addition, it was apparent that compared with GS115-Exmt (R145I), GS115-TLEI showed a lower decrease in the rate of the intracellular AOX activity. At 96 h, AOX activity of GS115-TLEI was present with a value of 68.37 ± 1.6 U·g^–1^ (Fig. 7D), which was 3.12-fold higher than that of GS115-Exmt (R145I) (Fig. 7C). Meanwhile, it could be found that no obvious intracellular amidase activity was detected in GS115-TLEI while the intracellular amidase activity of GS115-Exmt (R145I) was accumulated to 1.64 ± 0.13 U·ml^−1^ (Supplementary Fig. S7). The results suggested that it is desirable for improving cell activity and the secretory production of active trypsin by combining intramolecular modification and the self-activation strategy with N-terminal engineering. The mutant TLEI constructed here would have potential applications in production of trypsin at industrial scale.

In conclusion, although trypsin genes from different sources have been expressed in *P. pastoris*^7^,^42^,^43^ and the extracellularly expressed mature SGT was increased to 14.4 U·ml^−1^ (amidase activity) in 3-l fermenter^22^, the limits encountered are the protease toxicity of mature trypsin to the host cell and the instability because of self- autolysis. Therefore, development of a novel trypsin variant that can self-activate in secretory process is attractive. In the present study, inspired by the bovine trypsin activation mechanism and our previous results^17^, we constructed a number of variants and successfully achieved the secretory expression of trypsin with a self-activation strategy by fusion of an artificial peptide TrxA-GS*3-6H-D4K at the N-terminus of SGT. The site-directed mutagenesis of autolysis residue R145I was also engineered to improve stability against self-autolysis as well as catalytic efficiency. By combining these favorable factors, a self-catalyzed variant TLEI was constructed. In fed-batch fermentation, the production of the recombinant trypsin was significantly enhanced to 47.4 ± 1.2 U·ml^−1^ (amidase activity) with a productivity of 0.49 U·ml^−1^·h^−1^. In addition, the self-activation approach developed in this work could also be potentially used for production of other toxic enzymes.

## Methods

### Microorganisms and growth conditions

*Streptomyces griseus* ATCC 10137^TM^ which produced the SGT was purchased from the American Type Culture Collection (ATCC, USA). *P. pastoris* GS115 (His^−^) and plasmid pPIC9K were both purchased from the Invitrogen^TM^ (Beijing, China). The pMD19-T Sample vector was purchased from TAKARA (Dalian, China). Yeast nutrient medium, minimal dextrose medium, minimal medium, yeast extract peptone dextrose medium, buffered minimal glycerol-complex medium and buffered minimal methanol-complex medium were prepared by means of “*P. pastoris* expression Kit” (Pichia Multi-Copy Expression Kit, version A, Invitrogen BV, The Netherlands.).

### Plasmid construction and yeast transformation

The recombinant plasmids were constructed with the single strand oligonucleotide (Supplementary Table S1). As showed in Fig. 1, The DNA fragment of TLmt variants encoding α-factor signal peptide, differently artificial peptide and mature SGT were constructed by overlap extension PCR with corresponding primers contained conserved sequence (Supplementary Table S1). And the resultant fragment were inserted into pPIC9K plasmid, digested with *Bam*HI and *Not*I. Specially, the artificial peptide segment of TLmt (D4K) positioned between the C-terminus of the α-factor signal peptide and the N-terminus of SGT, which was composed of TrxA (thioredoxin, encoded by the *trxA* gene), GS*3 (GSGSGS, glycine-serine linker), 6H (His_6_-tag) and D4K (DDDDK, the partial bovine trypsinogen pro-peptide). In addition to the native bovine trypsinogen pro-peptide (D4K), other artificial peptides were also designed and comparatively studied (Fig. 1). The pPIC9K-*Exmt* (R145 mutations) and pPIC9K-*TLEI* were constructed by one step PCR according to Blunting Kination Ligation (BKL) Kit (TAKARA (TAKARA, Dalian, China)). pPIC9K-*Exmt* (R145 mutations) were amplified from previously constructed pPIC9K-*Exmt* plasmid with corresponding primers in (Supplementary Table S1), and pPIC9K-*Tlmt* (R145I) was constructed by primers Tlmt (R145I) 5 and Tlmt (R145I) 3 when pPIC9K-*Tlmt* (D4K) was used as temple. Then pPIC9K-*TLEI* was constructed from pPIC9K-*Tlmt* (R145I) with TLEI 5 and TLEI 3 primers. The constructed plasmids were transformed into *P. pastoris* GS115 (His^−^) by electroporation, according to *P. Pastoris* expression kit. The *P. Pastoris* strains harboring the variant *SGT* gene were screened in YPD plate with 4 mg·ml^−1^ geneticin.

### Expression and purification of different SGT variants

The *P. pastoris* transformant was cultured in 250 ml baffled flask supplied with 25 ml buffered glycerol-complex medium at 30 °C at an agitation of 250 rpm for 24 h. Then the cells were centrifuged and resuspended in 30 ml of buffered methanol-complex medium to an OD_600_ value of 1.0, and shaken at 30 °C at an agitation of 250 rpm in 250 ml baffled flasks for 144 h. The culture broth was supplemented with 1% (v/v) methanol every 24 h to induce the expression of recombinant SGT. All of variants were purified with the same procedures. The crude sample was concentrated by precipitation with 25–55% ammonium sulfate. Then the precipitate was resuspended in 5 ml buffer A (10 mM Tris–HCl, pH 8.0, 10% (w/v) glycerol, and 1 mM EDTA) and dialyzed overnight against 100 ml of the same buffer. The sample was loaded onto a Hitrap benzamidine FF column (Φ1.6 × 2.5 cm, GE Healthcare), previously equilibrated with buffer A. Then, the column was washed with buffer B (10 mM NaOAc, pH 5.0, 10% glycerol, 1 mM EDTA, and 0.5 M NaCl). Proteins were eluted with 6 M guanidinium chloride solution at a flow rate of 2.5 ml·min^−1^, and fractions containing trypsin activity were collected and dialyzed against buffer A^25^. TrxA was purified by HisTrap FF crude 5 ml (GE Healthcare) according to HisTrap column instructions. Protein concentration was estimated by a Coomassie Brilliant Blue R-250 binding assay, using a commercial standard protein solution containing bovine G-globulin. Protein concentration of effluents was monitored at 280 nm.

### SDS-PAGE analysis

The protein was fractionated by SDS-PAGE system. The samples (30 μl) were pretreated by mixing with 6 μl 5 × SDS-PAGE loading buffer in a 0.5 ml centrifugal tube. After vortexing for 5 seconds, the samples were incubated in 100 °C for 10 min, then each sample aliquot (10 μl) was loaded. Electrophoresis of the protein was performed on 12% separating gel with 5% stacking gel. The gels were kept at a constant voltage of 80 and 110 V at stacking and separating gel respectively, then stained with coomassie brilliant blue R-250. Sequencing five amino acids of N-terminal were done by Sangong Biotech Co., Ltd. (Shanghai, China).

### Determination of trypsin activity

The cultures of different SGT variants, were collected every 24 h and diluted to the same cell concentration with assay buffer. Then supernatant was prepared for measuring activity, by centrifuging culture solution at 10000g and 4 °C for 5 min, with triplicates measured in an effort to reduce error. The pellets were washed twice with assay buffer and then resuspended and disrupted by high pressure homogenization (Constant Cell Disruption Systems). The cell-free extract was used for measuring intracellular activity. Trypsin amidase activity was determined by spectrophotometer as the release of *p*-nitroanilide by hydrolysis of the artificial substrate N_α_-benzoyl-DL-arginine-*p*-nitroanilide (BAPNA)^44^. Briefly, sample (100 μl) was mixed with 800 μl of assay buffer (50 mM Tris-HCl, pH 8.0 at 37 °C) and 100 μl of 0.1 M BAPNA. The change in absorbance at 410 nm was monitored spectrophotometrically. One unit (U) of amidase was defined as the amount of enzyme required for producing a ΔA_410_ of 0. 1 per minute under the above conditions. The amidase activity of sample was calculated according to Equation 1:


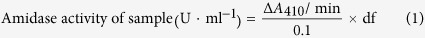


where df is the dilution factor.

Trypsin esterase activity was measured using N_α_-benzoyl-L-arginine ethyl ester hydrochloride (BAEE) as a substrate^45^. Immediately mix sample (200 μl) with 3 ml of assay buffer (67 mM, pH 7.6 sodium phosphate buffer containing 0.25 mM BAEE) at 25 °C. The change in absorbance at 253 nm was monitored spectrophotometrically. One unit (U) of esterase was defined as the amount of enzyme required for producing a ΔA_253_ of 0.001 per minute under the above conditions. The esterase activity of sample was calculated according to Equation 2:


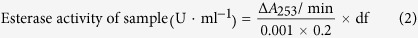


where df is the dilution factor; 0.2 means that 0.2 ml of sample was added into the reaction system.

### Molecular dynamics simulation and configuration of recombinant trypsin

The X-ray crystal structure of the wild type *Streptomyces* trypsin (PDB ID: 1SGT)^11^, downloaded from the RCSB Protein Data Bank (http://www.pdb.org/pdb/home/home.do), was used as the template for modeling. Stereo chemical analysis of the structure was performed using PROCHECK (http://nihserver.mbi.ucla.edu/SAVS/)^46^. The 3D model simulations were carried out by NAMD software with charm M force field (http://www.ks.uiuc.edu/Research/namd)^47^. Na^+^ was also added to neutralize the system. The pH 7.0 was set as default. Proteins were solvated in a cubic box consisting of TIP3P water molecules and the box size was chosen by the criterion that the distance of protein atoms was greater than 10.0 Å from the wall. A Ewald summation method was used for calculating the total electrostatic energy in a periodic box named Particle Mesh Ewald (PME). Structure minimization was performed to remove any unexpected coordinate collision and to get the local minima. The water box and the whole system were minimized using the descent method plus the conjugate gradient method^48^. After minimization, the system heating, equilibration and data sampling were carried out in turn. The system heating was performed gradually from 0 K to the desired temperature at a constant temperature and volume (NTV ensemble), followed by a further 150 ps simulation for equilibration and 3 ns or longer simulation for data sampling at constant temperature and constant pressure (NTP ensemble). The temperature was set as 300 K, 330 K respectively, at 1 atmospheric pressure. Structural diagrams were drawn using PyMol software (http://pym ol.sour ceforge.net/)^49^.

### Illustration of the simulated models

The simulated models of the trypsin variants have structural parameters from primary sequence to tertiary structure. Among these parameters, the amino acids, H bonds, Ion pairs, π-interactions (secondary structure parameters), and secondary structure contents (tertiary structural parameter) are important for the investigation. The amino acids can be calculated by the Vector NTI software^50^; H bonds were predicted by PyMol software (http://pymol.sourceforge.net/)^49^.

### Anti-autolysis comparison of SGT and recombinant SGT

Purified samples were diluted to same protein concentration (50 mg·l^−1^), then were incubated at 37 °C in dry bath for 15 days, with 1 ml enzyme solution in 1.5 ml eppendorf tube. The samples were collected to measure amidase activity every three days, initial activity defined as 100%. Triplicates were used to measure activity in an effort to reduce error.

### Determination of copy number of recombinant SGT (rSGT) gene by real-time quantitative PCR

The genomic DNAs of strains were prepared for absolute quantification using the TIANamp Yeast DNA Kit (Tiangen Biotech, Beijing, China). *GAPDH* (gene ID 8198905 which presents on the chromosome 2 of *P. pastoris*) was chosen as the reference gene. The pPIC9K-*rSGT* and pMD19-T-*GAPDH* were constructed to establish standard curves, and the amount of plasmid was measured using a Qubit^®^ 2.0 fluorometer (Life Technologies, USA). Real-time PCR primers were designed using Beacon Designer 7 software (Supplementary Table S1). The PCR mixture were prepared using SYBR^®^ Premix Ex TaqTM II (Tli RNaseH Plus) (TAKARA, Dalian, China), and amplification was performed using a Light Cycler^®^ 480 instrument (Roche Diagnostics Ltd., Rotkreuz, Switzerland). For each sample, reactions were performed in triplicates. Standard curves were generated by a linear regression of the mean Ct values plotted against log10 of diluted template. The copy number of the target gene was determined according to Equation 3^51^.


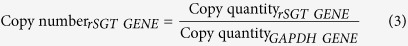


### Production of recombinant trypsin by fed-batch fermentation

After cultivating the cells at 30 °C and 200 rpm for 24 h in a 500 ml shake flask containing 50 ml YPD (20 g·l^−1^ glucose, 20 g·l^−1^ peptone, 10 g·l^−1^ yeast extract), 10% (v/v) of the inoculum was inoculated into a 3 l fermenter (LiFlus GM BioTRON, Korea) with 800 ml basal salts medium (BSM: 40 g·l^−1^ glycerol, 18 g·l^−1^ K_2_SO_4_, 4.13 g·l^−1^ KOH, 14.9 g·l^−1^ MgSO_4_·7H_2_O, 27 ml·l^−1^ H_3_PO_4_, 0.93 g·l^−1^ CaSO_4_) with 4.4 ml·l^−1^ PTM1 trace salts (PTM1: 6 g·l^−1^ CuSO_4_·5H_2_O, 0.09 g·l^−1^ KI, 3 g·l^−1^ MnSO_4_·H_2_O, 0.02 g·l^−1^ H_3_BO_3_, 0.2 g·l^−1^ MoNa_2_O_4_·2H_2_O, 0.5 g·l^−1^ CoCl_2_, 20 g·l^−1^ ZnCl_2_, 65 g·l^−1^ FeSO_4_·7H_2_O, 0.2 g·l^−1^ biotin, 5.0 ml·l^−1^ H_2_SO_4_). The pH of the medium was controlled at 5.5 with the addition of 50% ammonium hydroxide and 30% phosphoric acid. The temperature was controlled at 30 °C, and dissolved oxygen (DO) level was maintained over 30% of air saturation by a cascaded control of agitation rate (500–1000 rpm) and aeration rate (3–5 l·min^−1^). The fermentation process was composed of three phases. The fermentation began with glycerol batch phase until the initial glycerol was exhausted, as indication of DO level increased to more than 50%. In glycerol fed-batch phase, the feeding medium was pumped into the fermenter according to a predetermined protocol, which contained 50% (w/v) glycerol and 12 ml·l^−1^ PTM1 solution. Finally, the trypsin was expressed with induced by methanol in methanol fed-batch phase. Two hours after depletion of the glycerol with DO increased to more than 50% and dry cell weight (DCW) reached to 80 g·l^−1^, 1.8% (v/v) methanol containing 12 ml·l^−1^ PTM1 solution was fed with pH controlled at 5.5 and temperature controlled at 30 °C. The methanol concentration was maintained by the methanol on-line control station (FC2002, East China University of Science and Technology)^52^.

### Determination of cell concentration, residual glycerol, biomass and intracellular alcohol oxidase activity

Cell concentration was measured spectrophotometrically at 600 nm with the appropriate dilution. 10 ml sample was collected every 12 h, then centrifuged in a weighed centrifuge tube at 10,000 g and 4 °C for 10 min. The supernatant was prepared to detect the residual glycerol by HPLC with refractive index detector. The tubes containing the pellets were dried to constant weight at 80 °C for measuring DCW. Triplicates were used to determining activity in an effort to reduce error.

For intracellular alcohol oxidase (AOX) activity analysis, 1 ml sample was centrifuged under the above conditions. The pellets were washed twice with 50 mM phosphate buffer (pH 7.0), then resuspended in the same buffer and disrupted by high pressure homogenization (Constant Cell Disruption Systems). The cell-free extract was prepared to measure AOX activity by collecting centrifuged supernatant. Triplicates were used to determining activity in an effort to reduce error.

The intracellular AOX activity was assayed by measuring H_2_O_2_ produced during oxidation of methanol. The standard assay mixture contained 100 μM phosphate buffer (pH 7.0), 4.3 μM phenol, 15 IU peroxidase, 1 μM 4-aminoantipyrine, 400 μM methanol, and cell-free extract in a total volume of 3 ml. The reaction was carried out at 37 °C for 10 min, then the increase in absorbance at 500 nm was measured. One enzyme unit is defined as production of one μmol of H_2_O_2_ per minute^53^.

## Additional Information

**How to cite this article**: Zhang, Y. *et al.* Improved Production of Active *Streptomyces griseus* Trypsin with a Novel Auto-Catalyzed Strategy. *Sci. Rep.* **6**, 23158; doi: 10.1038/srep23158 (2016).

## Supplementary Material

Supplementary Information

## Figures and Tables

**Table 1:** Enzyme kinetics parameters of wild and recombinant SGT.

Variants	*K*_m_(mM)	BAPNA^a^*k*_cat_ (s^−1^)	*k*_cat_/*K*_m_(s^−1^·M^−1^)
BT	8.28 ± 0.33 × 10^−1^	5.03 ± 0.18 × 10^1^	6.08 ± 0.25 × 10^4^
SGT	7.88 ± 0.16 × 10^−2^	1.21 ± 0.42 × 10^3^	1.53 ± 0.05 × 10^7^
Exmt	3.05 ± 0.12 × 10^−2^	1.90 ± 0.10 × 10^3^	6.23 ± 0.32 × 10^7^
R145D	5.08 ± 0.30 × 10^−2^	3.58 ± 0.15 × 10^1^	7.07 ± 0.30 × 10^5^
R145I	3.42 ± 0.14 × 10^−2^	2.48 ± 0.10 × 10^3^	7.25 ± 0.43 × 10^7^
R145L	3.57 ± 0.18 × 10^−2^	1.35 ± 0.58 × 10^2^	3.80 ± 0.17 × 10^6^
R145M	4.14 ± 0.12 × 10^−2^	2.22 ± 0.08 × 10^2^	5.35 ± 0.17 × 10^6^
R145T	7.85 ± 0.31 × 10^−2^	2.47 ± 0.10 × 10^2^	3.15 ± 0.13 × 10^6^
R145V	9.20 ± 0.28 × 10^−2^	1.72 ± 0.07 × 10^1^	1.87 ± 0.10 × 10^6^

^a^The Lineweaver-Burk plots of substrate BAPNA was showed in Supplementary Fig. S8.
