# Supplementary material for: Improved Production of Active Streptomyces griseus Trypsin with a Novel Auto-Catalyzed Strategy
Source: Sci Rep. 2016 Mar 17;6:23158. doi: 10.1038/srep23158 (PMC4794721; doi:10.1038/srep23158)
Supplement: Supplementary Information [file srep23158-s1.pdf]

Supplemental information

Improved Production of Active *Streptomyces griseus* Trypsin with a Novel Auto-Catalyzed Strategy

Yunfeng Zhang [1, 2, 3], Zhenmin Ling [1], Zhen Kang [1, 2, 3], Jian Chen [2, 3] & Guocheng Du [2, 3]

[1]Key Laboratory of Industrial Biotechnology, Ministry of Education, Jiangnan University, 1800 Lihu Road, Wuxi, Jiangsu 214122, China; [2]Synergetic Innovation Center of Food Safety and Nutrition, 1800 Lihu Road, Wuxi, Jiangsu 214122, China; [3]School of Biotechnology, Jiangnan University, 1800 Lihu Road, Wuxi, Jiangsu 214122, China; Correspondence and requests for materials should be addressed to Z.K. (email: zkang@jiangnan.edu.cn) or G.C.D. (email: gcdu@jiangnan.edu.cn).

Supplementary Table1

Supplementary Table S1. Oligonucleotide primers used in this study

Name	Sequence of primers (5'→3')[a]
**Tlmt variants**	
TLmt-*α-factor* [b] 5	CGCGGATCCAAACGATGAGATTTCCTTCAATTTTTACTG
TLmt-*α-factor* 3	GGTGAATAATTTTATCGCTCATAGCTTCAGCCTCTCTTTTC
TLmt-*trxA* 5	GAAAAGAGAGGCTGAAGCTATGAGCGATAAAATTATTCACC
TLmt-*trxA* 3	AGAACCAGAACCGGAACCCGCCAGGTTAGCGTCGAGGAACTC
TLmt (D4K) 5	GGTTCCGGTTCTGGTTCTCATCACCATCACCATCACGATGACGA TGACAAGGTCGTCGGCGGAACCCGCG
TLmt (D4R) 5	GGTTCCGGTTCTGGTTCTCATCACCATCACCATCACGATGACGA TGACAGAGTCGTCGGCGGAACCCGCG
TLmt (D4D) 5	GGTTCCGGTTCTGGTTCTCATCACCATCACCATCACGATGACGA TGACGATGTCGTCGGCGGAACCCGCG
TLmt (APNP) 5	GGTTCCGGTTCTGGTTCTCATCACCATCACCATCACGCCCCCAA CCCCGTCGTCGGCGGAACCCGCG
TLmt (D4No) 5	GGTTCCGGTTCTGGTTCTCATCACCATCACCATCACGATGACGA TGACGTCGTCGGCGGAACCCGCG
TLmt (mutants)3	CCGGCGGCCGCTCAGAGCGTGCGGGCGGCCG
**Exmt (R145 mutations)**	
Exmt (R145D) 5	GATGAGGGCGGCAGCCAGCAGCGC
Exmt (R145I) 5	ATTGAGGGCGGCAGCCAGCAGCGC
Exmt (R145L) 5	TTGGAGGGCGGCAGCCAGCAGCGC
Exmt (R145M) 5	ATGGAGGGCGGCAGCCAGCAGCGC
Exmt (R145T) 5	ACTGAGGGCGGCAGCCAGCAGCGC
Exmt (R145V) 5	GTTGAGGGCGGCAGCCAGCAGCGC
Exmt (mutants) 3	GTTGGCGCCCCAGCCGGCGACGG
**TEI construction**	
TLmt (R145I) 5	ATTGAGGGCGGCAGCCAGCAGCGC
TLmt (R145I) 3	GTTGGCGCCCCAGCCGGCGACGG
TLEI 5	ATTGAGGGCGGCAGCCAGCAGCGC
TLEI 3	GTTGGCGCCCCAGCCGGCGACGG
**Real-time PCR**	
F_{rSGT}	ACACTGGTGGCGTTGATAC
R_{rSGT}	CGATGCCGACCTGAATCC
F_{GAPDH}	ACAAGGACTGGAGAGGTGGTAG
R_{GAPDH}	CGGTTGGGACACGGAAAGC

[a] Restriction sites were underlined

[b] α-factor was α-factor signal peptide

Supplementary Figures

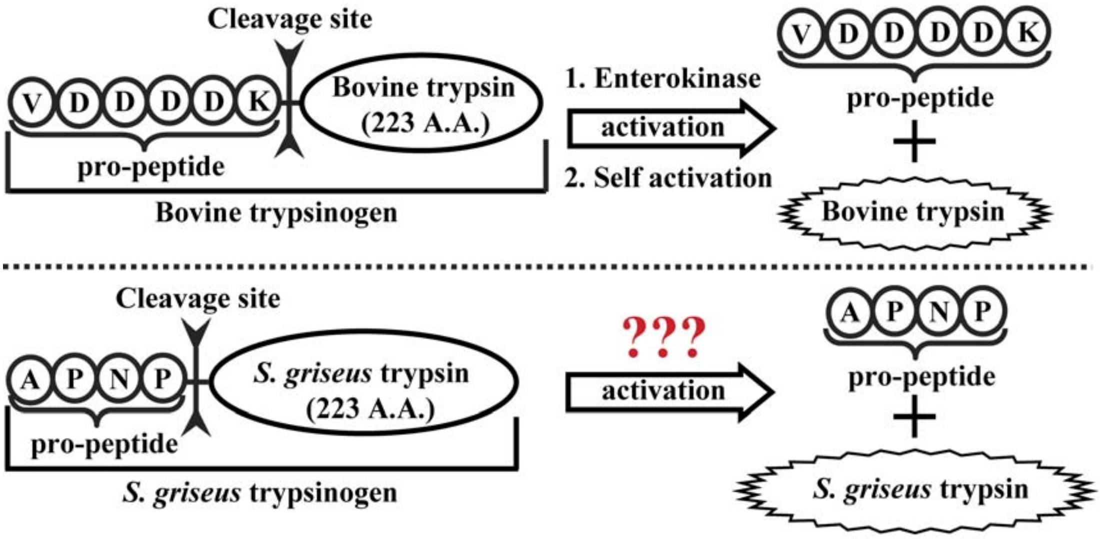

Supplementary Figure S1. Illustration of the different activation mechanism between BT and SGT. Bovine trypsinogen with partial pro-peptide VD4K, can be activated by enterokinase and slowly self-activate under optimal condition (pH, Tm, Ca^{2+} ion, etc.). Pro-peptide of *Streptomyces griseus* (*S. griseus*) trypsinogen (APNP), which can't be cleaved by trypsin, is removed when SGT is secreted in extracellular. So the activation mechanism of *S. griseus* trypsinogen is a puzzle.

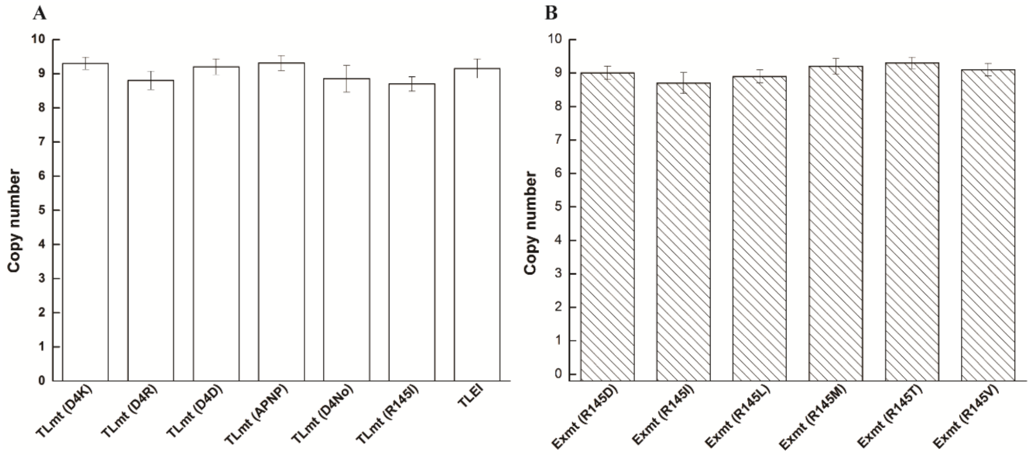

Supplementary Figure S2. Copy numbers of *SGT* gene on the chromosome. (A) The strains expressing SGT with different artificial peptides. (B) The strains expressing SGT with Exmt (R145 mutations). As showed in figure, all of the strains harbored 9 copies of recombinant *SGT* gene, screened with 4 mg·ml^{-1} geneticin on the YPD plate.

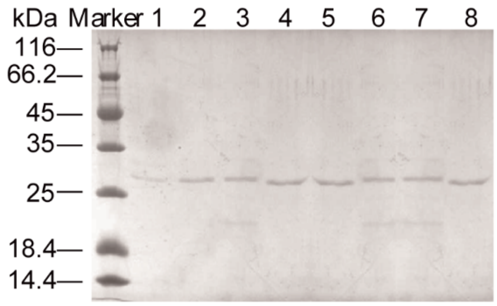

Supplementary Figure S3. SDS-PAGE of each purified Exmt (R145 mutations). 1, wSGT; 2, Exmt; 3, Exmt (R145D); 4, Exmt (R145I); 5, Exmt (R145L); 6, Exmt (R145M); 7, Exmt (R145T); 8, Exmt (R145V).

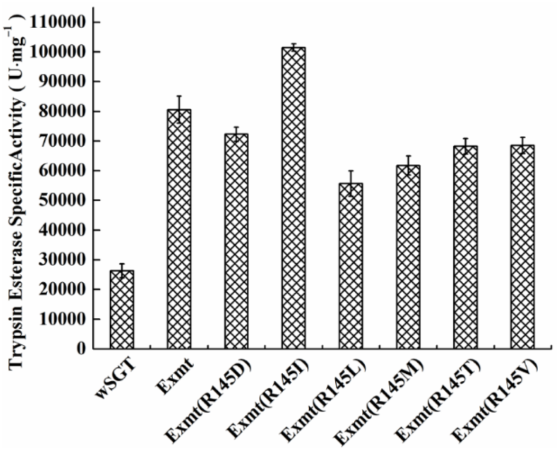

Supplementary Figure S4. Trypsin esterase specific activity of Exmt (R145 mutations). Exmt (R145I) had the highest esterase specific activity (101,491.58 ± 1,225.56 U·mg^{-1}).

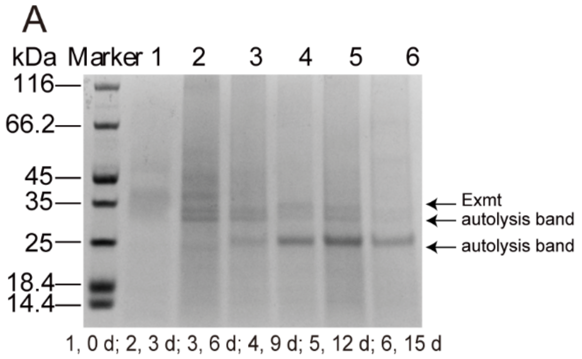
A
kDa Marker 1 2 3 4 5 6
116
66.2
45
35
25
18.4
14.4
Exmt
autolysis band
autolysis band
1, 0 d; 2, 3 d; 3, 6 d; 4, 9 d; 5, 12 d; 6, 15 d

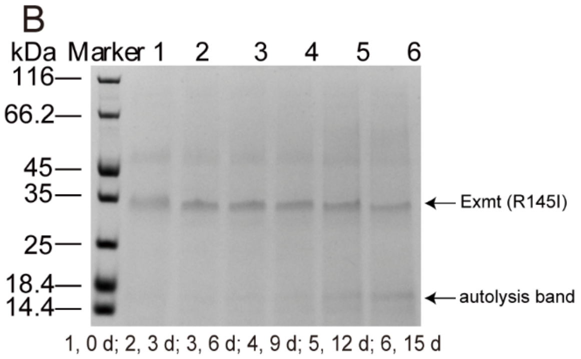
B
kDa Marker 1 2 3 4 5 6
116
66.2
45
35
25
18.4
14.4
Exmt (R145I)
autolysis band
1, 0 d; 2, 3 d; 3, 6 d; 4, 9 d; 5, 12 d; 6, 15 d

Supplementary Figure S5. SDS-PAGE of autolysis comparison. (A) Exmt was obviously degraded to lower molecular weight band by itself. (B) Exmt (R145I) mostly avoid self-autolysis.

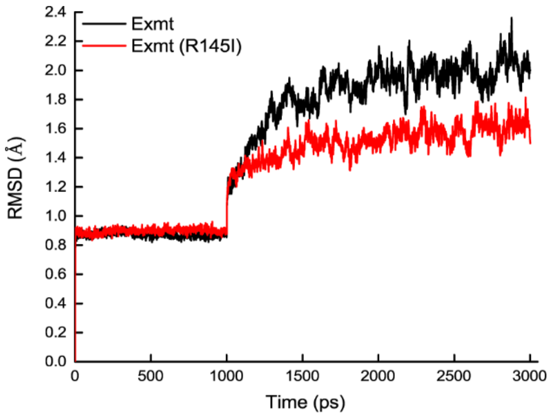

Supplementary Figure S6. RMSD (root mean square deviation) versus Time (ps) for Exmt and Exmt (R145I).

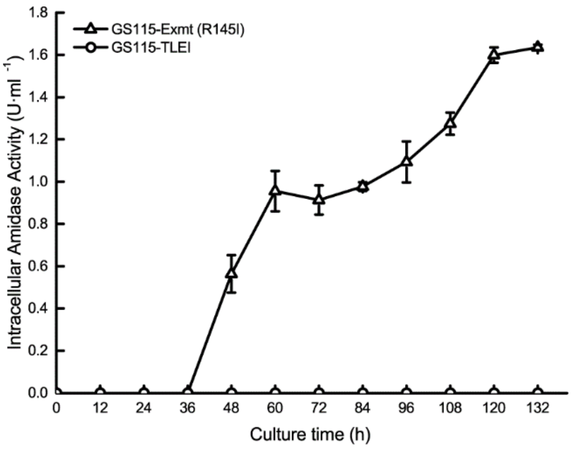

Supplementary Figure S7. Intracellular amidase activity of GS115-Exmt (R145I) and GS115-TLEI, in 3l fermenter. GS115-TLEI had no obvious intracellular activity by engineering N-terminus of SGT. While, intracellular amidase activity of GS115-Exmt (R145I) was 1.64 ± 0.13 U·ml^{-1}.

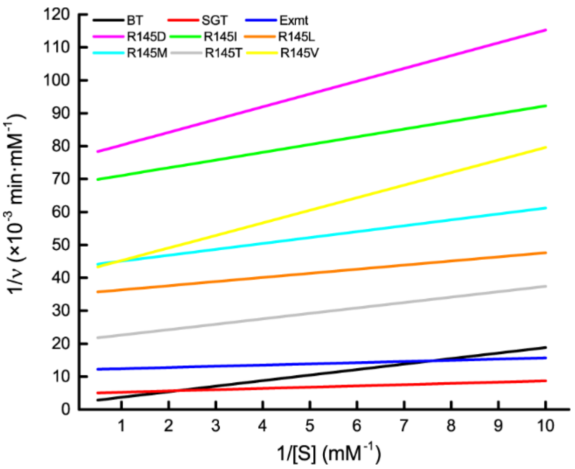

Supplementary Figure S8. Lineweaver-Burk plots of substrate BAPNA.
